# Effect of an educational intervention on HPV knowledge and vaccine attitudes among urban employed women and female undergraduate students in China: a cross-sectional study

**DOI:** 10.1186/1471-2458-13-916

**Published:** 2013-10-02

**Authors:** Irene J Chang, Rong Huang, Wei He, Shao-Kai Zhang, Shao-Ming Wang, Fang-Hui Zhao, Jennifer S Smith, You-Lin Qiao

**Affiliations:** 1 Department of Epidemiology, Cancer Institute of Chinese Academy of Medical Sciences, Peking Union Medical College, Beijing, P.R. China; 2 University of Miami Miller School of Medicine, Miami, FL, USA; 3 Gillings School of Global Public Health, University of North Carolina, Chapel Hill, NC, USA

**Keywords:** Cervical cancer, HPV education, Human papillomavirus, Prophylactic HPV vaccine, HPV knowledge, HPV attitudes, Vaccine acceptability

## Abstract

**Background:**

Due to the potential of human papillomavirus (HPV) vaccination for decreasing cervical cancer rates in Mainland China, where some of the highest incidences in the world have been reported**,** our study aimed to assess HPV and HPV vaccine knowledge, and to evaluate the effect of a brief educational intervention on HPV knowledge and vaccine acceptability in Chinese undergraduate students and employed women.

**Methods:**

This multi-center, cross-sectional study was conducted across five representative cities of the five main geographical regions of Mainland China. Participants were selected from one comprehensive university and three to four companies in each city for a total of six comprehensive universities and 16 companies. A 62-item questionnaire on HPV knowledge and HPV vaccine acceptability was administered to participants before and after an educational intervention. The intervention consisted of an informative group lecture.

**Results:**

A total of 1146 employed women and 557 female undergraduate students were surveyed between August and November 2011. Baseline HPV knowledge was low among both groups— 320/1146 (28%) of employed women and 66/557 (12%) of students had heard of HPV, while only 237/1146 (21%) of employed women and 40/557 (7.2%) of students knew that HPV is related to cervical cancer. After educational instruction, 947/1061 (89%) of employed women and 193/325 (59%) of students knew the relationship between HPV and cervical cancer (*χ*2 = 1041.8, p < 0.001 and *χ*2 = 278.5, p < 0.001, respectively). Post-intervention, vaccine acceptability increased from 881/1146 (77%) to 953/1061 (90%), (p = <0.001) in employed women and 405/557 (73%) in students to 266/325 (82%), (p < 0.001). Women in both groups cited concerns about the HPV vaccine’s safety, efficacy, and limited use to date as reasons for being unwilling to receive vaccination. 502/1146 (44%) of women were willing to vaccinate their children at baseline, which increased to 857/1061 (81%) post-intervention, p < 0.001.

**Conclusions:**

Incorporation of our lecture-based education initiative into a government-sponsored or school-based program may improve HPV-related knowledge and HPV vaccine acceptability. Further studies are needed to evaluate and standardize HPV education programs in China.

## Background

Despite recent strides made in HPV vaccination and education, over half a million women worldwide develop cervical cancer each year. Over 85% of these cases occur in developing countries such as China due to the lack of effective screening and prevention programs [1,2]. The incidence of cervical cancer in Mainland China increased from 5.14 persons per 100,000 in 2004 to 6.87 persons per 100,000 in 2008 [3-5]. Persistent infection with high-risk human papillomavirus (hrHPV) subtypes has been established as the primary cause of squamous cell cervical cancer [6]. In China, HPV 16 and 18 are implicated in the etiology of over 84.5% of cervical malignancies [7,8].

Both the bivalent and quadrivalent HPV vaccines have been demonstrated to effectively prevent the development of high-grade cervical neoplasias, particularly when given to girls before they become sexually active [9-12]. The prophylactic HPV vaccines currently undergoing clinical trials in Mainland China hold potential to significantly decrease cervical cancer mortality rates in the future. Prior to the licensure and widespread use of these vaccines, HPV education plays a crucial role in vaccine acceptability in the general population [13]. Studies have demonstrated that mothers with higher HPV knowledge have increased vaccine uptake and express greater willingness to vaccinate their children [14,15].

Previous studies on knowledge and attitudes about HPV among different groups of Chinese women have revealed high vaccine acceptability but low levels of knowledge about HPV and cervical cancer [16]. A pilot study by Kwan et al. (2008) demonstrated only 38% of women in Hong Kong had heard of HPV [17]. Zhao et al. (2012) confirmed that knowledge about HPV was similarly low in Mainland China, with 34% of urban women, 16% of rural women, and 44% of government officials having heard of HPV. Of concern, less than 20% of healthcare workers correctly identified the ideal time for HPV vaccination as prior to sexual debut [18]. These data suggest a strong need for HPV and HPV vaccine education in the general female population in Mainland China.

In recent years, there has been a proliferation of HPV education studies internationally. Educational instruction on HPV-related diseases has been demonstrated to effectively raise HPV knowledge and vaccine acceptability in America [19,20], Sweden [21], Korea [22], and Turkey [23,24]. Although previous studies have assessed the effect of education instruction on HPV-related knowledge in Hong Kong [25-27], the differing socioeconomic factors and the availability of the HPV vaccine in Hong Kong make the findings less applicable to Mainland China. In our present study, we report the results of a multi-center study examining the effect of a brief educational intervention on HPV vaccine-related knowledge and attitudes among employed women and undergraduate female students in Mainland China.

## Methods

### Participants and recruitment

This was a multi-center, cross-sectional study across the five main geographical regions of Mainland China. One large, economically developed city was selected from each of the five geographic regions of China (northern Beijing, eastern Hangzhou, central Changsha, southwestern Chengdu, and southern Guangzhou). Participants were then selected from one comprehensive university and three to four companies in each city for a total of six comprehensive universities and 16 companies. Participants were recruited by flyers and poster announcements in lecture halls, student centers, and the employment units at local companies.

### Informed consent

Prior to enrollment, all participants were fully informed about the objectives of the study, benefits and risks associated with participation, and assured of the confidentiality of the information they provide. After informed consent was obtained, a 62-item, multiple-choice questionnaire was administered to participants. The questionnaire was adapted from those based on expert opinions and used in previous studies among different populations across Mainland China [16,18]. The same questionnaire was administered again following the educational intervention to assess differences in HPV knowledge and vaccine attitudes. This study was approved by the Institutional Review Board of the Cancer Institute, Chinese Academy of Medical Sciences (CICAMS).

### Educational intervention

**Figure 1:**
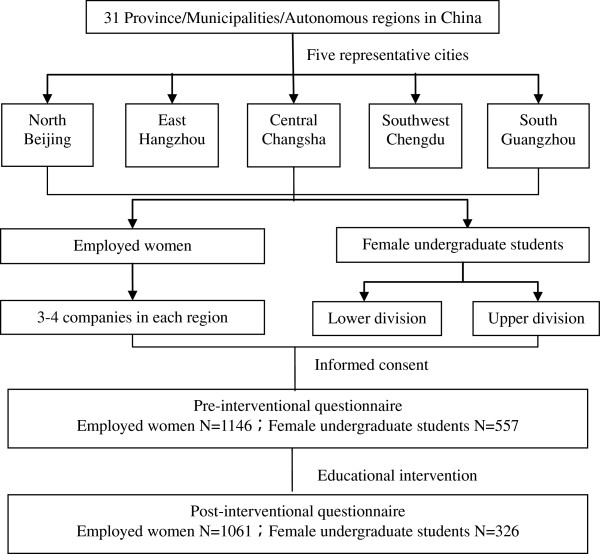
Study flowchart.

The educational intervention consisted of a one-hour group lecture followed by the same questionnaire as the one given after informed consent. Questions were written in Mandarin Chinese in simple terms appropriate for the level of education of the participants as previously validated [16,18]. Participants were encouraged to complete the questionnaire independently and trained staff members stood by to answer any questions participants had. The study flowchart is shown in Figure 1.

### Statistical analysis

The data manager organized the database using EpiData 3.1. To ensure accuracy, two different research members entered data separately. Data analysis was performed using SPSS software version 18.0. Chi-square or F-test analysis was performed on the demographic characteristics of participants such as age, occupation, and geographic region. Multivariate analysis of all descriptive factors was performed to compare pre- and post-interventional HPV and vaccine knowledge. Logistic regression was performed to analyze the association between potential predictor variables and willingness to receive vaccination. Odds ratios (ORs) with 95% confidence intervals (CIs) were calculated based on Wald Chi-square statistics. Statistical significance was set at p < 0.05.

## Results

From August to November of 2011,1146 employed women and 557 female undergraduate students were surveyed. After excluding incomplete questionnaires, 1061 and 325 surveys were included in the post-interventional analysis, respectively.

### Demographics

Table 1 shows demographic and medical information about the participants. Average age of 37.0 ± 10 for employed women and 20.4 ± 1.2 for undergraduate students. Among the undergraduate students, 385/557 (70%) were underclassmen and 163/557 (30%) were upperclassmen.

**Table 1:** Characteristics of employed women and female undergraduate students

**Characteristics**	**Employed women (n, %)**	**Undergraduate students (n, %)**
Total participants (N)	1146	557
Age (years, mean ± SD)	37.0 ± 10.0	20.4 ± 1.2
Age at menarche (years, mean ± SD)	13.6 ± 13.0	13.1 ± 1.3
**Education level**		
< High school	408 (35.6)	-
≥ Some college	703 (61.3)	-
University grade*		
Lower classmen	-	385 (69.1)
Upper classmen	-	163 (29.3)
**Number of lifetime sexual partners**		
1	668 (58.3)	-
≥2	87 (7.6)	-
**Contraceptive method**		
None	520 (45.3)	-
Condoms (barrier method)	438 (38.2)	-
**Undergoes regular gynecologic exam**		
Yes	868 (75.7)	-
No	178 (15.5)	-
**History of genital tract infections**		
Yes	408 (35.6)	-
No	524 (45.7)	-
**Obstetric history**		
Nulliparous	425 (37.1)	-
1 live birth	664 (57.9)	-
≥ 2 live births	57 (5.0)	-
**Regions**		
North/Beijing	245 (21.4)	197 (35.4)
East/Hangzhou	248 (21.6)	64 (11.5)
Central/Changsha	229 (20.0)	33 (5.9)
Southwest/Chengdu	176 (15.4)	67 (12.0)
South/Guangzhou	248 (21.6)	196 (35.2)

*Underclassmen refers to first- and second-year undergraduate students, upperclassmen refers to third- and fourth-year undergraduate students.

### Pre-intervention HPV- related knowledge

**Figure 2:**
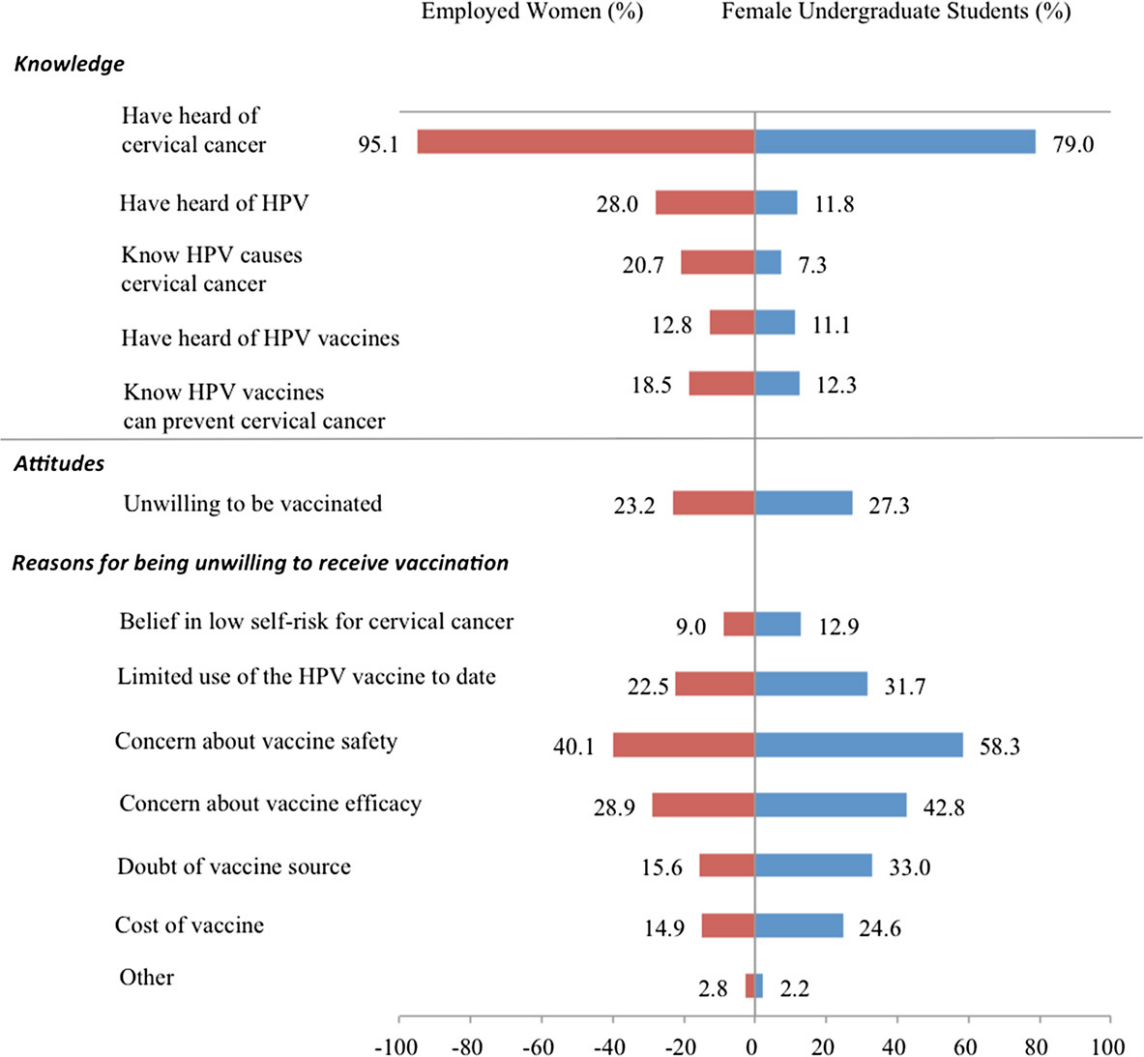
Baseline HPV, cervical cancer, and HPV vaccine knowledge.

At baseline, 1089/1146 (95%) of employed women and 440/557 (79%) of female undergraduate students had heard of cervical cancer. However, HPV knowledge was low in both groups, with only 321/1146 (28%) of employed women and 66/557 (12%) female students having heard of HPV. 237/1146 (21%) of employed women and 40/557 (7.2%) of students knew that HPV infection causes cervical cancer, but only 51/1146 (4.5%) and 18/557 (3.2%) knew the association between HPV and genital warts, respectively. Similarly, only 147/1146 (13%) employed women and 62/557 (11%) students had heard of the HPV vaccine. 212/1146 (18%) and 69/557 (12%) knew that the vaccine could prevent cervical cancer, respectively. 103/1146 (9%) of employed women were not concerned about developing cervical cancer. Knowledge of HPV and cervical cancer did not differ significantly between women from different geographical regions (p > 0.05). Baseline HPV knowledge is summarized in Figure 2.

### Post-interventional HPV-related knowledge

**Figure 3:**
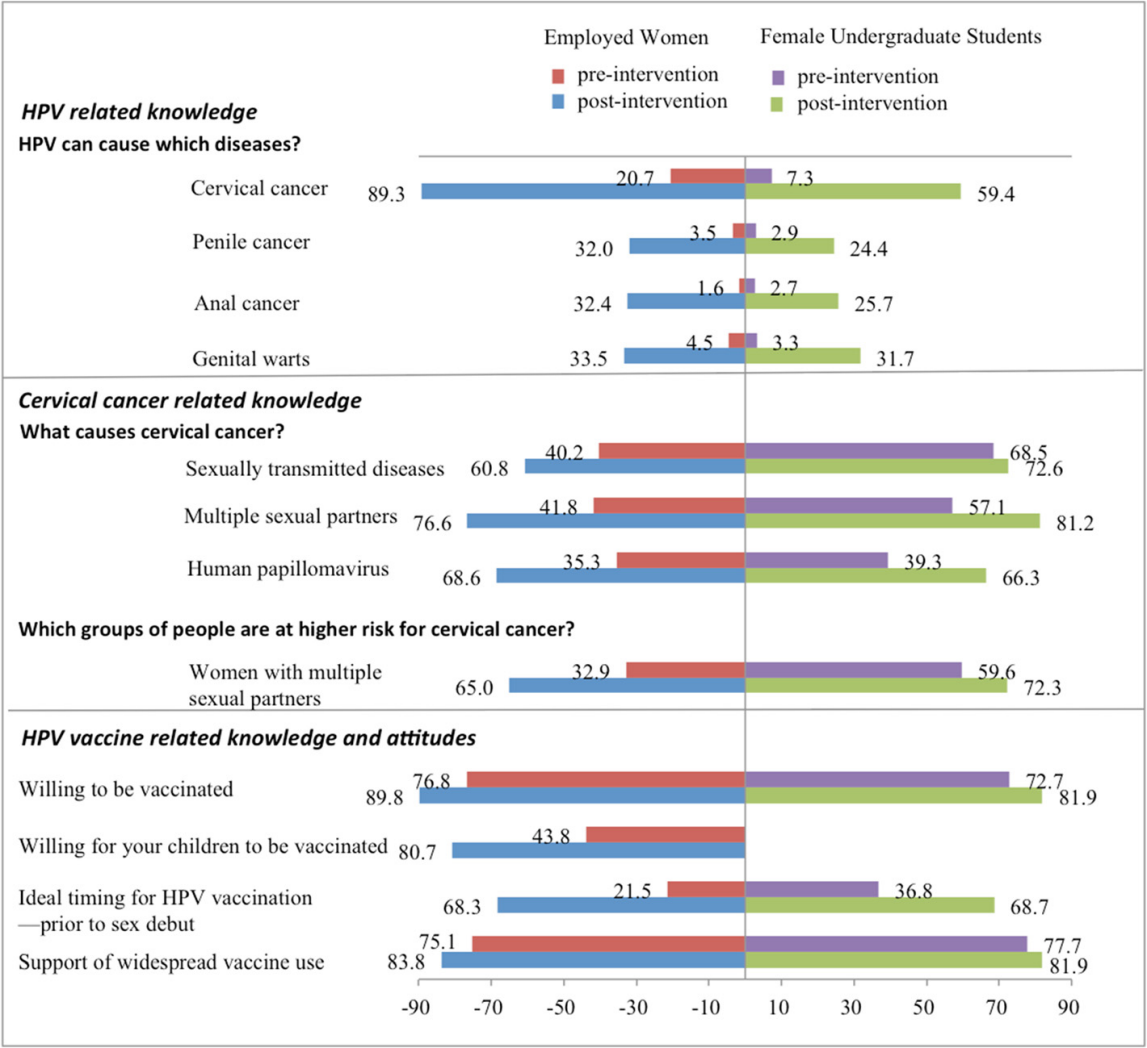
Comparison of pre- and post-interventional HPV-related knowledge.

After a brief educational narrative about HPV and cervical cancer, HPV-related knowledge increased significantly. Comparison of pre- and post-interventional HPV knowledge is shown in Figure 3. Post-intervention, 947/1061 (89%) of employed women knew that HPV infection caused cervical cancer from 237/1146 (21%) at baseline, a 4.2 fold increase from baseline (*χ*^*2*^ = 1041.8, p < 0.001). Among undergraduate students, knowledge of this causal relationship increased over seven-fold from 40/557 (7.2%) to 193/325 (59%), (*χ*^*2*^ = 278.5, p < 0.001).

Post-interventional knowledge about HPV-related diseases and risk factors for HPV infection also increased significantly. In particular, correct identification of the ideal time for HPV vaccination as prior to sexual debut increased significantly from 246/1146 (21%) to 725/1061 (68%) in employed women and 205/557 (37%) to 223/325 (69%), (*χ*^*2*^ = 79.6, p < 0.001) in female students. Employed women also reported increased intention to undergo routine gynecologic exam after the intervention from 601/1146 (52%) to 974/1061 (92%), (p < 0.001).

### Attitudes and acceptability towards the HPV vaccine

Willingness to receive HPV vaccination increased significantly post-intervention from 881/1146 (77%) to 953/1061 (90%) in employed women and from 405/557 (73%) to 266/325 (82%) in students, (p < 0.01). Female undergraduate students over age 20 were 1.6 times more willing to receive vaccination (0.9-2.9) than those under age 20. Knowledge of cervical cancer, concern about contracting cervical cancer, and approval of widespread usage of the vaccine were factors that influenced willingness to receive vaccination (OR 2.4 (1.3-4.8), 2.8 (1.6-5.0), and 4.3 (2.4-7.6), respectively). 265/1146 (23%) of employed women and 152/557 (27%) of female undergraduate students were unwilling to receive vaccination for themselves. The most common reasons against vaccination given by both groups of participants was concern about vaccine safety (325/1146 (58%) in women and 459/557 (40%) in students), concern about vaccine efficacy (331/1146 (29%) in women and 238/557 (43%) in students), and limited usage of the vaccine to date (258/1146 (23%) in women and 177/557 (32%) in students). Baseline attitudes and vaccine acceptability are shown in Figure 2 and comparison of pre- and post-interventional vaccine acceptability are shown in Figure 3.

Post-intervention, willingness of employed women to vaccinate their children increased from 502/1146 (44%) to 857/1061 (81%), (*χ*^*2*^ = 261.7, p < 0.001). However, 175/1061 (16%) women were still unwilling to vaccinate their children after educational instruction due to concerns about vaccine safety (249/1061 (24%)), concerns about vaccine efficacy (136/1061 (13%)), and belief that their children are too young to be at risk (233/1061 (22%)). Comparison of pre- and post-educational HPV vaccine attitudes is shown in Figure 3.

Logistic regression examining the effect of various sociodemographic predictors and cervical cancer risk on maternal willingness to vaccinate their children is presented in Table 2. Women were more likely to be willing to vaccinate their children if they were between the ages of 36 to 45 (OR = 0.35 (0.18-0.68), p = 0.002) and if they were concerned about contracting cervical cancer themselves (OR = 1.8 (1.1-3.2), p = 0.03). Overall, 902/1703 (53%) of all participants chose the China Center for Disease Control and 426/1703 (25%) chose women and children’s hospitals as the ideal location to receive the HPV vaccine.

**Table 2:** Logistic regression analysis for maternal willingness to vaccinate their children

**Characteristics of employed women**	**N**	**Willingness to vaccinate their children**			
		**N (%)**	**Ajusted OR (95% CI)**	**Walds *χ*2**	**P**
Age (yrs)					
≤25	164	61 (59.2)	1.00		
26-35	292	91 (37.1)	1.16 (0.37-3.7)	0.06	0.80
36-45	282	96 (41.2)	0.35 (0.18-0.68)	9.45	**0.00**
>45	253	99 (51.3)	0.46 (0.25-0.87)	5.76	0.02
Education level					
≤High school	408	154 (49.36)	1.00		
> High school	703	229 (41.11)	0.93 (0.53-1.6)	0.07	0.79
Occupation					
Skilled professionals	821	278 (42.25)	1.00		
Manual laborers	132	53 (51.46)	0.52 (0.16-1.8)	1.10	0.29
Others	64	25 (50.00)	1.2 (0.29-4.9)	0.05	0.82
Household registration					
Rural	112	39 (50.65)	1.00		
Urban	707	241 (43.11)	0.91 (0.29-2.9)	0.02	0.88
History of childbirth					
No	524	172 (41.55)	1.00		
Yes	408	159 (46.90)	0.98 (0.6-1.6)	0.01	0.94
Number of lifetime sexual partners					
1	668	231 (41.77)	1.00		
≥2	87	43 (54.43)	2.0 (0.95-4.2)	3.3	0.1
Prior experience with vaccinations outside the EPI (e.g., flu vaccine)					
No	810	279 (42.27)	1.0		
Yes	266	100 (47.39)	1.0 (0.58-1.7)	<0.001	1.0
Fear of developing cervical cancer					
No	222	62 (35.23)	1.0		
Yes	586	243 (49.90)	1.8 (1.06-3.2)	4.6	**0.0**
Voluntarily sought out HPV-related information					
No	981	344 (42.95)	1.0		
Yes	82	39 (55.71)	1.4 (0.64-3.2)	0.8	0.4

## Discussion

The brief educational intervention in our study effectively raised HPV and HPV vaccine knowledge in undergraduate students and employed women, and also increased vaccine acceptability. Post-intervention, women were twice as likely to report willingness to vaccinate their children and correctly identified younger ages as ideal for receiving the HPV vaccine. This is of particular significance because a recent study suggests for optimal vaccine compliance, girls in China should be vaccinated between the ages of 13-15, prior to concluding their mandatory schooling and becoming sexually active [18].

While the HPV vaccine may reduce the incidence of cervical cancer in the future, clinical trials across China are ongoing. An effective HPV education program that addresses specific knowledge gaps and common questions among women of different regions, education levels, and cultures has been demonstrated to increase the success of large-scale vaccination programs [21]. Studies in the U.S. have found that educational programs should emphasize vaccine effectiveness and the high likelihood of HPV infection, and address barriers to vaccination [14]. A similar study in Japan found that vaccine safety and cost should be addressed to increase maternal vaccine acceptability for their children [28]. Women also reported increased vaccine acceptability for themselves and their children after hearing physician and government endorsements of the HPV vaccine [25,28].

To our knowledge, the present study represents the first evaluation of a large-scale HPV educational intervention in Mainland China. Kwan et al. [26,27] have conducted similar HPV knowledge and education studies in Hong Kong, yet no similar educational interventions have been conducted in Mainland China. A previous study by Zhang and colleagues [18] examined HPV knowledge alone in China among the general female population, healthcare personnel, and government officials. Our results confirm their findings of low baseline HPV knowledge among employed women and female undergraduate students. In addition, our survey of employed women and female undergraduate students paints a more comprehensive picture of HPV knowledge in different female population groups across Mainland China.

The results of our present study indicate that baseline HPV knowledge is low among employed women and female undergraduate students in northern, eastern, central, southern, and southwestern regions of China. Through our study, we also gained valuable insight into the preferred methods of obtaining HPV-related information and social beliefs held among Chinese women. Employed women and female college students preferred to receive HPV-related information via school or hospital-based lectures. They also believed that a national organization such as the Center for Disease Control should be responsible for providing the vaccine. In addition, vaccine safety, efficacy, and cost remained concerns and barriers to vaccination. Women had no preference for either the domestic or foreign HPV vaccine, but believed that the government should support and partially subsidize the cost. Future HPV education programs can focus on addressing these aforementioned issues. Furthermore, over 76% of employed females believe that getting vaccinated is a societal responsibility that should be shared by both genders. Thus, a government-sponsored, school-based education program can effectively disperse safe intercourse practices and HPV vaccine information to both male and female students.

Strengths of our study include the relatively large population sampled across five main geographical regions of Mainland China. We also targeted employed women and undergraduate female students, two populations who have not been surveyed before. Our findings are consistent with previous studies, which found that HPV knowledge and vaccine acceptability were higher among college-educated women [18,25].

Limitations of our study include the cross-sectional design. While our educational intervention significantly increased post-test HPV knowledge and vaccine acceptance, the strength of causality is limited by the absence of a control group. Also, although our questionnaire was adapted from previous studies, we did not apply a scale to assess its validity. Future large-scale studies should devise a reliability scale to evaluate the questionnaire. Furthermore, our study was mainly carried out in urban centers among women with higher education levels thus due to the potential sampling bias, our findings may not be generalized to the entire Chinese population. HPV knowledge and vaccine receptivity have been shown to vary widely based on region, sociodemographic factors and education level [14,16]. In 2007, a survey of 400 Uyghur women in Xinjiang found only 5% had heard of cervical cancer [29]. A more recent study in 2011 on 1005 Han, Uyghur, and Muslim women residing in Xinjiang found that only 5.2% of Uyghur women and 11.5% of Muslim women knew the association between HPV infection and cervical cancer, suggesting that the findings of our study may not be generalized to different groups of Chinese women [30]. This highlights the need for culturally salient HPV and cervical cancer education especially among Chinese ethnic minority populations. Furthermore, while HPV and HPV vaccine knowledge significantly improved immediately following the brief educational intervention, its lasting impact is unknown. It is also uncertain whether vaccine acceptability and positive attitudes towards vaccination would persist and translate into higher vaccination rates.

## Conclusions

Our study demonstrates that incorporation of our educational initiative into a government sponsored or school based program may improve willingness to accept HPV vaccination. The potential implications of our study are far-reaching during this pivotal time of HPV vaccine clinical trials in China. It is important to establish the link between HPV infection and the development of cervical cancer, and to educate women on the ideal time to vaccinate their children. Studies have shown that maternal attitudes towards vaccination greatly impacts vaccination rates among children. It is also imperative to dispel common beliefs that only women with multiple sexual partners need to be vaccinated.

Future directions for research include the development of a longitudinal, national HPV education program appropriate for school-based curriculum. Due to our limited participant sampling, our findings may not be generalized to the entire heterogeneous Chinese female population. Widespread implementation among different geographic regions and minority populations across China would shed light on the long-lasting impact of HPV education. Also, it is important develop evaluation and feedback tools to assess the effectiveness of the program. Lastly, follow-up studies must be performed to see if increased HPV and HPV vaccine knowledge translates into higher vaccination rates and vaccine compliance within China.

## Competing interests

The authors declare that they have no competing interests.

## Authors’ contributions

IJC participated in data analysis and drafted the manuscript. FHZ and YLQ conceived of the design and coordination of the study. RH, WH and SKZ performed the statistical analysis. SMW and RH participated in the data collection. FZ guided the statistical analysis and revisions of the manuscript. All authors read and approved the final manuscript.

## Pre-publication history

The pre-publication history for this paper can be accessed here:

http://www.biomedcentral.com/1471-2458/13/916/prepub
